# 573. AIMS-trained Residents Exhibit Specific Communication Skills During Virtual Encounters with Standardized Vaccine-Hesitant Parents Following an Online Training Program

**DOI:** 10.1093/ofid/ofac492.626

**Published:** 2022-12-15

**Authors:** Shanna M Barton, Aaron W Calhoun, Carrie A Bohnert, Sara Multerer, Victoria A Statler, Gary S Marshall

**Affiliations:** Norton Children's and University of Louisville School of Medicine, Louisville, Kentucky; University of Louisville, Louisville, Kentucky; University of Louisville School of Medicine, Louisville, Kentucky; University of Louisville, Louisville, Kentucky; Norton Children's and University of Louisville, Louisville, Kentucky; Norton Children's and University of Louisville School of Medicine, Louisville, Kentucky

## Abstract

**Background:**

There are no accepted best practices for counseling vaccine-hesitant parents, and training in this area is not required in residency. In a prior study (*J Pediatr* 2022;241:203-11), we demonstrated that in-person training in a structured communication strategy called AIMS (Announce, Inquire, Mirror, Secure) resulted in behaviors of interest during live encounters with standardized patients (SPs) portraying vaccine-hesitant parents. We investigated whether similar effects would be seen if training and SP encounters occurred in a virtual environment.

**Methods:**

Pediatrics and Medicine-Pediatrics residents were randomized to receive either AIMS or control training. Subjects underwent pre- and post-training SP encounters simulating an immunization visit for a 4-month-old. SP case materials were modified to more closely approximate well-intentioned reluctance to vaccinate and allow for more authentic interaction. Encounters were video-recorded and assessed by 3 raters using the Vaccine Hesitancy Communication Assessment (VHCA), developed and characterized in the initial study but modified based on factor analysis to improve reliability and validity. Subject confidence and SP evaluations of the encounter were assessed pre- and post-training. Investigators, subjects, SPs, and video raters were blinded to treatment allocation.

**Results:**

Fifty-three subjects completed the protocol and 47 had complete video files. Subject confidence improved in both groups (Panel A). No differences in SP evaluations were detected between groups (B). Preliminary analysis demonstrated that AIMS behaviors were more commonly detected among AIMS-trained subjects than control, as evidenced by an increase in VHCA score (C).

**Figure:**
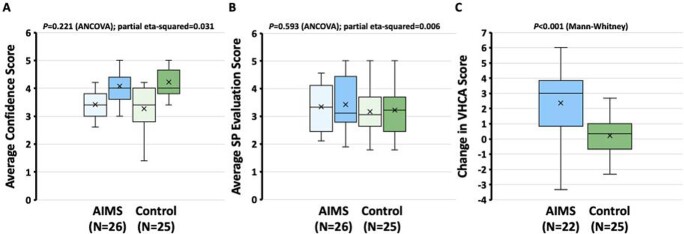

**Conclusion:**

Communication training and assessment using SPs were both successfully transitioned to a virtual environment; this opens the possibility of efficient training and assessment of residents who are not located on site. Training increased confidence non-specifically. Encounters with SPs can serve as a model to detect learned vaccine-specific communication behaviors among resident providers, but post-encounter assessments by SPs remained insensitive to differences in those behaviors despite modification of the case materials.

**Disclosures:**

**Shanna M. Barton, MD, M.Sc.**, Sanofi Pasteur: Grant/Research Support **Aaron W. Calhoun, MD, FSSH**, Sanofi-Pasteur: Grant/Research Support|Society for Simulation in Healthcare: Board Member|Society for Simulation in Healthcare: Honoraria **Victoria A. Statler, M.D., M.Sc.**, Astellas: University Research Support|Gilead: University Research Support|Pfizer: Advisor/Consultant|Sanofi: University Research Support|Seqirus: Advisor/Consultant **Gary S. Marshall, MD**, GlaxoSmithKline: Advisor/Consultant|GlaxoSmithKline: Grant/Research Support|GlaxoSmithKline: Honoraria|Merck: Advisor/Consultant|Merck: Grant/Research Support|Merck: Honoraria|Pfizer: Advisor/Consultant|Pfizer: Grant/Research Support|Pfizer: Honoraria|Sanofi: Advisor/Consultant|Sanofi: Grant/Research Support|Sanofi: Honoraria|Seqirus: Advisor/Consultant|Seqirus: Grant/Research Support|Seqirus: Honoraria.

